# Determinants of Public Attitudes towards Euthanasia in Adults and Physician-Assisted Death in Neonates in Austria: A National Survey

**DOI:** 10.1371/journal.pone.0124320

**Published:** 2015-04-23

**Authors:** Erwin Stolz, Nathalie Burkert, Franziska Großschädl, Éva Rásky, Willibald J. Stronegger, Wolfgang Freidl

**Affiliations:** Institute of Social Medicine and Epidemiology, Medical University of Graz, Graz, Austria; Medical University of Vienna, AUSTRIA

## Abstract

**Background:**

Euthanasia remains a controversial topic in both public discourses and legislation. Although some determinants of acceptance of euthanasia and physician-assisted death have been identified in previous studies, there is still a shortage of information whether different forms of euthanasia are supported by the same or different sub-populations and whether authoritarian personality dispositions are linked to attitudes towards euthanasia.

**Methods:**

A large, representative face-to-face survey was conducted in Austria in 2014 (n = 1,971). Respondents faced three scenarios of euthanasia and one of physician assisted death differing regarding the level of specificity, voluntariness and subject, requiring either approval or rejection: (1) abstract description of euthanasia, (2) abstract description of physician-assisted suicide, (3) the case of euthanasia of a terminally-ill 79-year old cancer patient, and (4) the case of non-voluntary, physician assisted death of a severely disabled or ill neonate. A number of potential determinants for rejection ordered in three categories (socio-demographic, personal experience, orientations) including authoritarianism were tested via multiple logistic regression analyses.

**Results:**

Rejection was highest in the case of the neonate (69%) and lowest for the case of the older cancer patient (35%). A consistent negative impact of religiosity on the acceptance across all scenarios and differential effects for socio-economic status, area of residence, religious confession, liberalism, and authoritarianism were found. Individuals with a stronger authoritarian personality disposition were more likely to reject physician-assisted suicide for adults but at the same time also more likely to approve of physician-assisted death of a disabled neonate.

**Conclusion:**

Euthanasia in adults was supported by a partially different sub-population than assisted death of disabled neonates.

## Introduction

Euthanasia remains a controversial topic in both public and scientific discourses in Europe. Over the last two decades, acts of legalisation in several Western European countries [1–2] and increasing acceptance in the general public associated with societal changes in terms of educational attainment, secularisation, and increased appreciation of individual liberties [3–5] have spurred polarisation regarding a ‘good death’ against the background of higher life expectancy, increased risk of functional impairment in old age and chronic disease but also medical advancements regarding artificial life extension. ‘Euthanasia’ in this context refers to the voluntarily, patient-requested, deliberate ending of life by a medical professional administering a lethal substance. As a subcategory, ‘physician-assisted suicide’ refers to a situation, where an individual takes his or her own life but requires the help of a physician [6]. In contrast, the term ‘physician-assisted death’ can also include an in- or non-voluntary life-ending without explicit request, e.g. in case of an unbearably suffering infant, who cannot communicate a wish to die.

Currently, only a few studies on attitudes towards euthanasia in adults are based on representative surveys of the general population, whereas virtually none exist regarding assisted death of neonates or infants with the exception of one small-scale study based on a convenience sample [7]. The acceptance of euthanasia and assisted death both among the general public and medical staff has been reported to be lower when it comes to younger, non-terminally ill, or incompetent patients [8–12] and patient requests were found to be the most important factor in the evaluation of acts of euthanasia [13–14]. Furthermore, previous studies have consistently identified the level of education as a positive and religiosity as a negative empirical correlate of acceptance of euthanasia in adults [5, 15–24]. The first effect has been repeatedly attributed to more liberal orientations and more distinct desire for self-determination of higher educated individuals (“free choice” argument). The latter is attributed to the rejection of euthanasia by religious authorities and their teachings, rooted in religious beliefs of absolute morals and the divine privilege of giving and taking lives (“sanctity of life” argument) [25], adherence to key religious beliefs such as eternal damnation for (assisted) suicide or heavenly salvation as a future relief for those who suffer, and integration into religious social networks, reinforcing these beliefs [26]. Mixed results have been obtained for gender, age, and area of residence (urban vs. rural) [5, 17, 19–21]. Recently, it has been suggested that personal experience in end-of-life-care for family members or friends is associated with higher support for euthanasia in the face of grave and in the end futile suffering [24], whereas a reversed argument has been brought forward for physicians, who due to occupational responsibility, involvement in the actual act and potential legal consequences are more reserved towards euthanasia than the general public [10, 12, 24, 27–28]. Only few studies, however, assessed whether these determinants vary between different types and measurements of euthanasia [12, 24, 29] and none compared physician-assisted death of neonates to scenarios of euthanasia so far. Furthermore, the psychological concept of authoritarianism—which describes an individual’s disposition towards submission to religious and moral authorities, social conformism and hostility against social deviance [30]—has rarely been linked to attitudes towards euthanasia. The historical roots of the concept—the occupation with antidemocratic personality dispositions [31], which assumingly have co-enabled the gruesome non- and involuntary euthanasia of more than 250,000 ‘[…] children and adults whose lives were considered unworthy of living’ ([32], p. 109) in Austria [33–35]—and the incompatibility of authoritarian personality dispositions with the ‘[…] notion of self-determination and individual control in matters of life and death’ ([36], p. 2615) are, however, indicative. In our analysis, we consider three original aspects of authoritarianism: anxiety in the face of new and unknown situations and developments, social conformism, and hostility against those deviating from social norms. In Austria, killing on request and assisted suicide are illegal (§77 and §78 Austrian Criminal Code) and both acts are clearly rejected by traditional and religious authorities. We therefore expect that persons with an authoritarian disposition tend to oppose euthanasia as illegitimate life-ending decision of adults. In the case of a severely disabled neonate, expected to live in poor life quality for several years—category three according to the Groeningen Protocol [37]—however, a reversed relationship with authoritarianism is expected since individuals with strong authoritarian personality traits are characterised by a limited empathy for weaker members of society (S2 Table) and hostility toward deviation from what is considered as ‘normal’ or ‘natural’ [30], both applying in the case of the severely ill or disabled neonate.

The aim of our study, therefore, was to investigate whether determinants of approval vary by subject (older person vs. neonate), specificity (abstract vs. specific) and by whether a person consciously requested death or not (voluntary vs. non-voluntary).

## Data and Methods

A cross-sectional omnibus survey based on a representative sample of the Austrian population aged 15 years and older using a stratified random sampling procedure was conducted in early 2014. Strata were based on federal state, district and municipality size. Households for the sample were drawn randomly within these strata proportional to the actual number of households. Within households, individuals were selected using the Kish-Selection-Grid. Computer-assisted personal interviews (CAPI) were carried out by the Institute of Empirical Social Research (IFES, Vienna) at the behest of the authors. At a response rate of 47.5%, 1,971 interviews were completed (51.4% females; mean age = 47.5 years/SD = 18.5). Weights were calculated based on population data from Statistics Austria (2013) in order to ensure representativity of the sample for the Austrian population aged 15 years and more. Minimal differences between the weighted and unweight data suggest a high sample quality. Acceptance and rejection of euthanasia and physician-assisted death was measured via four different scenarios (S1 Table). In the first scenario, the respondents were confronted with a case of requested euthanasia, in the second scenario with a case of requested assisted suicide. Both referred to unspecified individuals, i.e. remained abstract. The third scenario presented a more specific situation of requested euthanasia of an older, terminally-ill cancer patient and the last scenario described a case of non-voluntary physician-assisted death of a severely ill or disabled new-born. Read out answer categories included only ‘for it’ or ‘against it’ or else ‘yes’ and ‘no’ in order to encourage a definitive statement in the context of a challenging ethical question.

The survey segment on personal experiences included items on whether the respondent has knowledge about legalisation of euthanasia abroad (yes/no), has personal experience in caring for seriously ill individuals (yes/no) or in end-of-life care (yes/no) and whether they work in the health professions (yes/no). The section ‘orientations’ comprises the variables political orientation (left/centre/right), socio-cultural orientation (strongly/rather/rather not /not at all liberal) and the level of religiosity (strongly/rather /rather not /not at all religious). As for authoritarianism, measurements relying on items of behaviour and experience [38] were chosen as indicators (S2 Table). Authoritarianism was calculated as a unit-value index, i.e. the sum-score for each person was divided by the number of valid answers in the eight Likert-scaled items upon the successful testing for internal consistency (Cronbach’s alpha = 0.79) and one-dimensionality by means of Factor Analysis (S2 Table). Authoritarianism ranged from one (strong) to four (not at all) and showed a skew to the left, i.e. the majority of Austrians indicated weak authoritarian traits. Authoritarianism showed a weak negative correlation with liberalism (r = -0.23, p>0.001) and does not correlate with the level of religiosity (r = -0.03, p = 0.137). In addition, we collected socio-demographic information such as gender, religious confession, age, socio-economic status, area of residence, health status (very good/good/moderate/poor/very poor), small children in the household (yes/no) and household size (see Table 1 for details). Socio-economic status (SES) was constructed from three variables: Level of formal education (compulsory schooling to completed higher education, 6 categories), household income (<450 € to >3,900 €, 20 categories), and occupation of the head of the household (13 categories). The categories of each variable correspond to a validated national scoring system (e.g. compulsory schooling = 20 points, higher education = 140 points). SES categories from A (high) to E (low) resulted from the respondent’s position within the aggregate index range (> 90% = A, 90–70% = B, 70–30% = C, 30–10% = D, < 10% = E) [39].

**Table 1:** Sample characteristics.

Variable	N	%
**Gender**		
Male	920	46.7
Female	1051	53.3
**Age**		
15–24 years	178	9.0
24–34 years	383	19.4
35–44 years	315	16.0
45–59 years	557	28.3
60–74 years	405	20.5
75+ years	133	6.7
**Socio-economic status**		
A (high)	200	10.1
B	425	21.6
C	786	39.9
D	365	18.5
E (low)	195	9.9
**Area of residence**		
Rural/Village	782	39.7
Town	516	26.2
City	258	13.1
Metropolis	415	21.1
**Confession**		
Catholic	1415	72.6
Protestant	88	4.5
Muslim	71	3.6
Other	54	2.8
No Confession	320	16.4
**Household size**		
Single	682	34.6
2 Persons	699	35.5
3+ Persons	590	29.9
**Total**	1971	100

Unweight data.

All data analyses were performed using IBM SPSS Statistics 21.0 for windows and R: A language and environment for statistical computing 2.15. Pearson’s chi-squared test was used to assess bivariate relationships and logistic regression analyses were used to model rejection of euthanasia and physician- assisted death in the four given scenarios. To ensure comparability of effect sizes across models irrespective of potentially varying unobserved heterogeneity, odds ratios were double-checked with average marginal effects (not shown) and no divergences were found. Nonlinearity regarding authoritarianism in the fourth scenario (neonate) was modelled using two natural splines (knot = 3).

### Ethical approval

The study was carried out in compliance with the principles laid down in the Helsinki Declaration. No children were included in the study sample. Selected households received a written invitation to participate in the survey including details regarding topics covered and length of the interview. Households were contacted a few days later by IFES (Institute of Empirical Research Vienna) by telephone describing again topic, length and required information regarding the survey, ensured anonymity of all personal data and obtained consent for arranging a face-to-face interview. Respondents were selected randomly within the household (Kish-Selection-Grid) and verbal, informed consent by the selected participant is obtained again on-site. In case of the selection of 15–17 year-olds, verbal informed consent was instead obtained from a legal guardian. If no such consent was given, the interview was not conducted. Additionally, interviews could be terminated by the respondent at any point. Therefore, completed interviews document the informed consent of participants. The entrusted agency (IFES) conducting the interviews is bound to the code of conduct and standards of both EUROQUEST and the World Association of Opinion and Marketing Research Professionals (ESOMAR). The consent procedure described above and the conductance of this study were approved by the Ethics Committee of the Medical University of Graz (EK-number 26–425 ex 13/14).

## Results

### Descriptive Analysis

**Fig 1:**
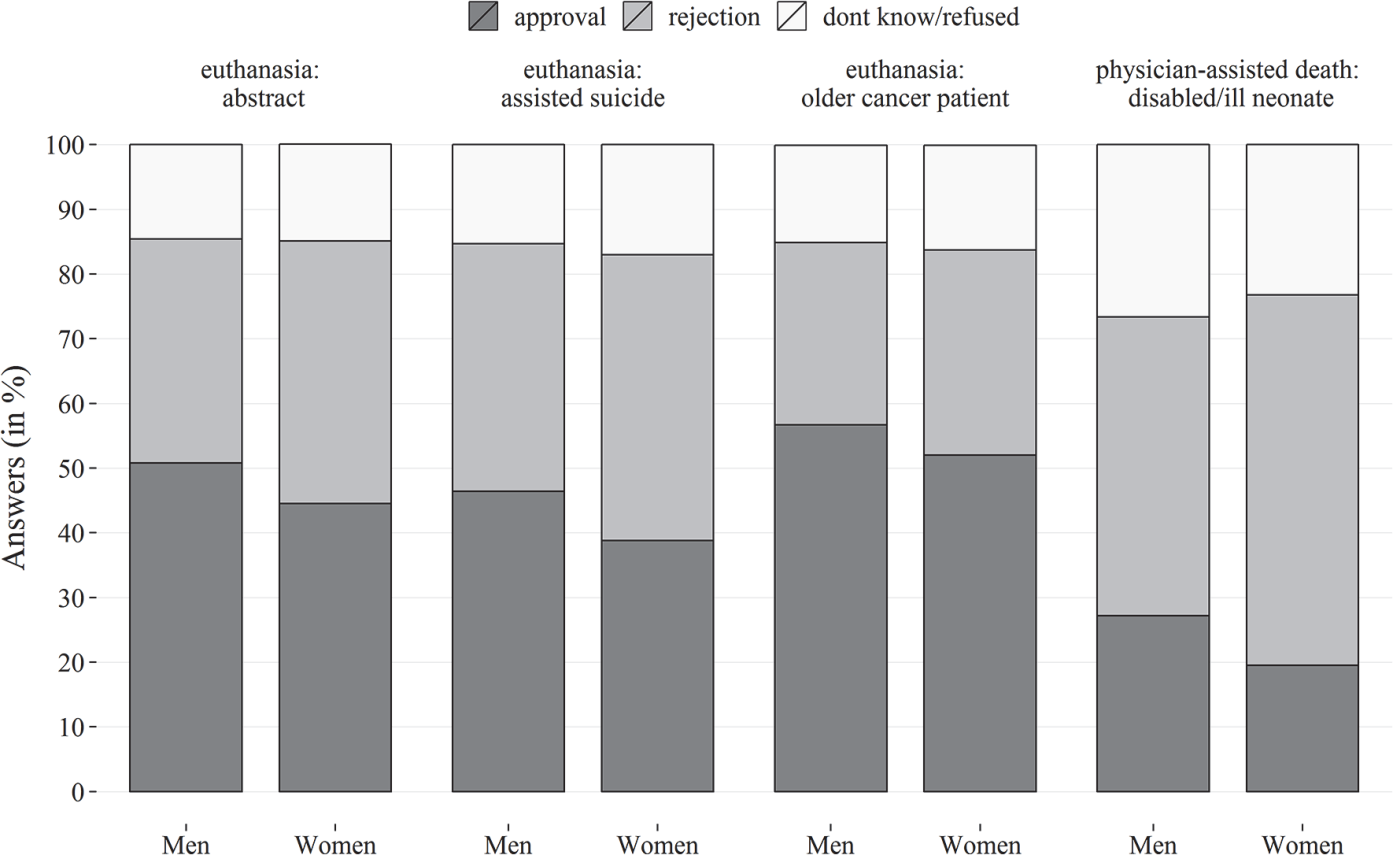
Prevalence of approval and rejection in four scenarios of euthanasia and assisted death in Austria. Weighted data, n = 1999/2000, own calculations.

Rejection rates concerning euthanasia and physician-assisted death varied across the scenarios (see Fig 1). A narrow majority of Austrians approved of euthanasia in the case of the suffering older cancer patient who asks for a lethal injection. Support for abstractly formulated euthanasia and physician-assisted suicide ranged between 43% and 48%, with men being more likely in favour of all specified types of euthanasia. Approval was lowest in the scenario of non-voluntary physician-assisted death for the severely disabled or ill neonate, supported by more than a quarter of the surveyed men and a fifth of the surveyed women. The proportion of those abstaining from a decisive answer was substantial and varied considerably. 15%–17% failed to give a definitive statement regarding euthanasia, and 27% of the male and 23% of the female population abstained from a clear choice in the case of physician-assisted death of the neonate. Within the valid answers, differences in approval across the four scenarios were even more pronounced: Physician-assisted death of the neonate: 30.9%, abstract, physician-assisted suicide: 50.7%, abstract euthanasia: 55.8% and specific case of euthanasia of older cancer patient: 64.4%. Overall, those on the margins of political (left wing/right wing), religious (very/not at all), socio-cultural orientations (very liberal/not at all liberal) and authoritarian disposition (strong/weak) seemed more determined in their answering behaviour and less likely to abstain from either a clear approval or rejection of euthanasia or physician-assisted death compared to those individuals located in the more moderate centre. The four outcome variables correlated positively with each other, but again indicated a clear line of demarcation between the case of the neonate and the three scenarios of euthanasia depicting adults. Among the latter, significant correlation coefficients ranged from 0.52 to 0.67, while approval in the case of the neonate was somewhat weaker correlated (r = 0.37–0.43) with approval of euthanasia.

**Fig 2:**
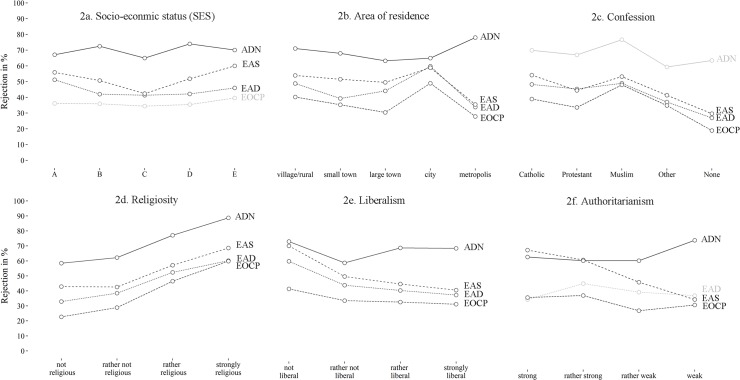
Bivariate analyses of rejection of euthanasia and assisted death in four scenarios for selected predictors. Weighted data, n = 1999/2000, own calculations, ADN: specific scenario of non-voluntary physician-assisted death of a severely ill or disabled neonate; EOCP: specific scenario of euthanasia of older cancer patient; EAS: abstract scenario of physician-assisted suicide; EAD: abstract scenario of euthanasia, darker lines represent significant associations (< 0.05) according to Pearson Chi² test, lighter lines represent non-significant results. Authoritarianism was categorised as follows: 1.00–1.75 = strong, 1.76–2.50 = rather strong, 2.51–3.25 = rather weak, 3.26–4.00 = weak.

Fig 2 shows bivariate statistics concerning the rejection in the different scenarios for selected predictors. The socio-economic position shows a curvilinear association with abstract euthanasia and assisted suicide (2a) insofar as people in both the highest 10% and the lowest 10% tended to disapprove more often than those in the middle. People in the capital Vienna (metropolis) approved more often, while those in provincial cities were more likely to reject euthanasia compared to those living in rural areas and Vienna, except in the case of assisted death of the neonate, where the relationship was reversed (2b). People without religious confession were more open towards euthanasia than people from all major denominations within Austria, whereas religious confession remained non-significant regarding the neonate (2c). Substantial differences in terms of rejection levels among strongly to not at all religious persons amounted to approximately 20–30% in all scenarios (2d). In the case of the older cancer patient, for example, 22.7% of the non-religious Austrians rejected an act of euthanasia, in comparison to 59.8% of their strongly religious counterparts (p<0.001). The association between liberalism and attitudes towards euthanasia and assisted death yielded corresponding results (2e), yet of less linear fashion, smaller magnitude and deviation regarding the physician-assisted death of the neonate. For authoritarianism, finally, both mean differences and using categories (2f) indicated a clear negative relationship regarding assisted suicide (p<0.001), i.e. those rejecting assisted suicide showed more pronounced authoritarian traits. A less pronounced negative pattern was visible regarding the older cancer patient (p = 0.039). Finally, a positive association was implied regarding the neonate (p<0.001), i.e. people with strong or at least some authoritarian characteristics were less likely to reject and more likely to approve of physician-assisted death of the severely disabled or ill neonate compared to those without such a disposition.

### Multivariate Analysis

Multiple logistic regression analyses mostly confirmed the results of the bivariate analyses aforementioned and particularly highlighted the role of political, religious, socio-cultural orientations and authoritarianism vis-à-vis the socio-demographic determinants and the role of personal experiences (Table 2). Nested models including all three blocks of independent variables (socio-demographics (a), knowledge and experience (b), and orientations (c)) showed the largest, significant improvements in model fit when adding the set of orientations (e.g. abstract euthanasia: AIC-a: 1972; AIC-ab: 1966, p_a/ab_ = 0.009; AIC-abc: 1939, p_ab/abc_ <0.001). Nagelkerke’s pseudo-R² indicated that the present set of predictors is able to explain a substantial amount of variance in all four models.

**Table 2:** Binary logistic regression analyses of rejection of euthanasia and physician-assisted death.

	Euthanasia									Physician-assisted death		
	Abstract euthanasia			Abstract assisted suicide			Specific older cancer patient			Specific disabled/ill neonate		
	OR	(CI)	p-value	OR	(CI)	p-value	OR	(CI)	p-value	OR	(CI)	p-value
**SOCIO-DEMOGRAPHICS**												
**Gender (ref: male)**												
Female	1.13	(0.90–1.41)	0.307	**1.32**	**(1.05–1.66)**	**0.017**	0.94	(0.74–1.19)	0.590	**1.50**	**(1.16–1.94)**	**0.002**
**Age (ref: 15–24 yrs.)**												
24–34 yrs.	1.58	(1.00–2.53)	0.054	1.13	(0.71–1.79)	0.607	1.08	(0.66–1.76)	0.762	1.19	(0.71–1.97)	0.513
35–44 yrs.	1.57	(0.98–2.53)	0.064	1.26	(0.78–2.03)	0.343	1.20	(0.73–1.99)	0.487	1.51	(0.90–2.54)	0.118
45–59 yrs.	**1.64**	**(1.04–2.62)**	**0.036**	1.30	(0.82–2.05)	0.267	1.04	(0.65–1.69)	0.871	1.16	(0.70–1.91)	0.568
60–74 yrs.	**2.05**	**(1.23–3.44)**	**0.006**	1.17	(0.70–1.97)	0.540	1.19	(0.70–2.04)	0.522	1.35	(0.76–2.38)	0.302
75+ yrs.	1.62	(0.86–3.08)	0.137	1.02	(0.53–1.94)	0.963	0.94	(0.49–1.82)	0.857	1.65	(0.80–3.45)	0.176
**SES (ref: E low)**												
A (high)	0.76	(0.43–1.33)	0.336	1.18	(0.67–2.08)	0.566	1.09	(0.60–1.96)	0.784	**0.47**	**(0.25–0.87)**	**0.017**
B	**0.57**	**(0.35–0.92)**	**0.022**	0.96	(0.58–1.57)	0.871	1.13	(0.68–1.90)	0.633	0.65	(0.37–1.12)	0.125
C	**0.58**	**(0.37–0.90)**	**0.015**	**0.54**	**(0.34–0.84)**	**0.007**	1.02	(0.64–1.62)	0.940	**0.50**	**(0.30–0.81)**	**0.006**
D	**0.55**	**(0.35–0.87)**	**0.011**	0.76	(0.47–1.20)	0.234	0.80	(0.50–1.30)	0.367	0.97	(0.58–1.63)	0.922
**Residence area (ref: Rural/Village)**												
Town	1.21	(0.92–1.60)	0.182	0.97	(0.73–1.28)	0.836	0.84	(0.63–1.13)	0.259	0.93	(0.68–1.27)	0.642
City	**1.87**	**(1.32–2.65)**	**<0.001**	1.11	(0.79–1.57)	0.549	**1.70**	**(1.20–2.41)**	**0.003**	0.96	(0.66–1.40)	0.843
Metropolis	0.84	(0.61–1.16)	0.282	**0.58**	**(0.42–0.80)**	**0.001**	0.77	(0.44–1.32)	0.146	**1.77**	**(1.21–2.62)**	**0.004**
**Confession (ref: Catholic)**												
Protestant	0.85	(0.50–1.41)	0.524	0.78	(0.45–1.34)	0.364	0.77	(0.55–1.09)	0.347	0.86	(0.47–1.60)	0.617
Muslim	1.21	(0.64–2.30)	0.560	1.63	(0.87–3.14)	0.133	**2.16**	**(1.11–4.27)**	**0.024**	1.16	(0.57–2.51)	0.688
Other	0.76	(0.37–1.51)	0.435	0.80	(0.38–1.62)	0.534	0.84	(0.39–1.75)	0.650	0.59	(0.28–1.28)	0.171
None	**0.57**	**(0.40–0.81)**	**0.002**	**0.57**	**(0.40–0.80)**	**0.001**	**0.57**	**(0.38–0.83)**	**0.004**	0.91	(0.62–1.34)	0.623
**State of health (very good to very poor)**	0.99	(0.85–1.15)	0.891	1.09	(0.93–1.36)	0.285	0.93	(0.80–1.09)	0.390	**0.80**	**(0.67–0.95)**	**0.012**
**Small child in household (ref: no)**												
Yes	1.12	(0.69–1.80)	0.653	**1.64**	**(1.01–2.68)**	**0.046**	1.16	(0.70–1.90)	0.562	1.81	(1.00–3.45)	0.058
**Household size (ref: living alone)**												
2 persons	1.07	(0.80–1.44)	0.647	0.88	(0.66–1.19)	0.412	0.81	(0.59–1.11)	0.183	**1.73**	**(1.23–2.43)**	**0.002**
3+ persons	1.35	(0.94–1.93)	0.099	0.84	(0.59–1.21)	0.362	0.81	(0.55–1.18)	0.274	**1.68**	**(1.12–2.53)**	**0.013**
**PERSONAL EXPERIENCE**												
**Knowledge of euthanasia legislation in NL? (ref: no)**												
Yes	**0.57**	**(0.40–0.81)**	**0.002**	**0.69**	**(0.48–0.99)**	**0.046**	**0.69**	**(0.48–1.00)**	**0.049**	0.81	(0.54–1.20)	0.289
**Caring for ill person (ref: no)**												
Yes	1.21	(0.88–1.69)	0.244	1.16	(0.83–1.63)	0.376	1.18	(0.83–1.66)	0.350	1.01	(0.69–1.47)	0.960
**Caring for dying person (ref: no)**												
Yes	0.88	(0.63–1.22)	0.431	0.78	(0.56–1.10	0.161	0.80	(0.56–1.13)	0.199	1.32	(0.91–1.93)	0.147
**Work in the health sector (ref: no)**												
Yes	1.11	(0.67–1.84)	0.692	1.05	(0.61–1.81)	0.863	1.42	(0.82–2.48)	0.212	1.33	(0.73–2.54)	0.368
**ORIENTATIONS**												
**Political position (ref: centre)**												
Left	1.28	(0.98–1.69)	0.075	**1.36**	**(1.03–1.81)**	**0.030**	**1.37**	**(1.03–1.80)**	**0.032**	1.21	(0.89–1.67)	0.230
Right	**1.35**	**(1.01–1.82)**	**0.044**	**1.35**	**(1.01–1.82)**	**0.047**	1.08	(0.79–1.47)	0.624	1.25	(0.89–1.75)	0.202
**Religiosity (strong to weak)**	**0.68**	**(0.58–0.78)**	**<0.001**	**0.76**	**(0.65–0.88)**	**<0.001**	**0.53**	**(0.45–0.62)**	**<0.001**	**0.68**	**(0.57–0.80)**	**<0.001**
**Liberalism (strong to weak)**	**1.22**	**(1.03–1.45)**	**0.023**	**1.22**	**(1.02–1.45)**	**0.029**	1.08	(0.91–1.30)	0.380	1.14	(0.94–1.38)	0.182
**Authoritarianism (strong to weak)**	1.12	(0.92–1.38)	0.265	**0.61**	**(0.49–0.75)**	**<0.001**	1.02	(0.83–1.27)	0.824	0.51	(0.08–2.89)	0.452
**Spline (2)**										**3.69**	**(2.21–6.20)**	**<0.001**
**Constant**	0.94			1.26			0.90			3.01		
**Pseudo R² (Nagelkerke)**	0.129			0.150			0.139			0.156		
**AIC**	1939			1899			1795			1560		
**N**	1474			1450			1449			1309		

Unweight data; OR = odds ratios, (CI) = 95% confidence intervals; AIC = Akaike Information Criterion; religiosity, liberalism and authoritarianism entered as continuous variables; authoritarianism in physician-assisted death of the neonate was modelled non-linearly using two natural splines (knot = value 3), allowing for two separate slopes (see also Fig 2), Spline (2) refers to the second slope.

When controlling for the aforementioned ideological convictions, gender remained statistically significant only for physician-assisted suicide and physician-assisted death of the neonate. SES proved to be curvilinearly associated with rejection of euthanasia in the cases of abstract euthanasia and assisted suicide, indicating that disapproval was higher at both the upper and lower ends of the Austrian social ladder compared to the middle classes. In the case of the neonate, it was not only the middle class (C) that showed a lower probability to reject euthanasia (OR = 0.50, p = 0.006) compared to the lowest social tier (E) but also the highest social class (OR = 0.47, p = 0.017). Multivariate results for location and religious confession are in line with those from the bivariate analysis. Having a small child in the household indicating (grand-)parenthood increased the odds of rejecting assisted suicide and living in larger household decreased the odds of approving regarding assisted death of the severely ill or disabled neonate.

Knowledge about the legalisation of euthanasia in the Netherlands decreased rejection of euthanasia in adults in all three scenarios, whereas it seemed irrelevant in the neonate’s case. Multiple regression analyses show that actual experience with caring for seriously ill or dying persons, or working in the health sector did not independently correlate with the attitudes towards euthanasia or physician-assisted death.

The positioning on the political spectrum (right, centre or left) showed only small effects regarding euthanasia in adults insofar as individuals in the political centre tended to be slightly more permissive than those on the left or right spectrum, whereas no significant associations show for the neonate’s case. Religiosity on the other hand proved highly significant across all regression models and consistently showed the same effect: the higher the reported level of religiosity, the higher was the likelihood of rejecting any form of euthanasia or physician-assisted death of the neonate. Furthermore, each increase regarding liberalism is associated with a 22% increase in the odds of approving of abstractly described euthanasia or assisted suicide, whereas no statistically significant relation was found for socio-cultural orientations in the more descriptive scenarios of the older cancer patient and the severely ill or disabled neonate. While no significant effect for authoritarianism was visible concerning abstract euthanasia and (after controlling for other variables) for the older cancer patient scenario, a higher degree of authoritarianism corresponded to a lower chance approving of assisted suicide, meaning that for each change in the three-stage variable authoritarianism, we expect to see a 64% increase in the odds of rejecting assisted suicide (p<0.001). In comparison, the direction of the association was reversed in the case of the disabled or ill neonate and followed a non-linear pattern. A decrease of the level of authoritarianism (between highly authoritarian and rather not authoritarian) did at first not correspond with a change in rejection but sharply increased (OR = 3.69, p>0.001) within the higher region of the scale, i.e. among those reporting lower levels of authoritarianism (see also Fig 2).

## Discussion

The difference in rejection between the specific and non-voluntary physician-assisted death of the neonate and the three scenarios of (voluntary) euthanasia in adults, the difference in the proportion of non-valid answers, and the differences in determinants of the multivariate analyses—where for characteristics in several variables, higher acceptance of euthanasia goes in hand with lower acceptance of assisted death of the neonate—suggest that these represent two different social and ethical issues. The lower rate of approval of the neonate’s death and the differential effects in the multivariate model likely reflect the young age, and the lack of control and autonomy of the neonate compared to euthanasia in (older) adults able to make decisions. Euthanasia is part of an established public discourse and as such seems least controversial in the case of the older cancer patient in pain requesting it, while no such discussion or polarised and publicly supportable opinion exists for the non-voluntary assisted death of severely ill or disabled neonates. The inhibition threshold of approving the death of the neonate should therefore be substantially higher, particularly taking into consideration the tabooed crimes against disabled children and adults of the Nazi regime in Austria (1938–1945). Nevertheless, a considerable minority of more than 20% of the respondents in face-to-face interviews supported actively ending the neonates’ live in the face of a shortened life span characterised by severe disability and a low overall quality.

The multivariate analyses showed two consistent findings across the models. Firstly, a personal experience with caring for ill or dying persons or working in the health sector did not show any significant, independent effect regarding the approval or rejection of euthanasia or assisted death. This could be attributable to the fact that the character and the course of events within the care-taking experience could lead people to be both more inclined to accept such acts, e.g. in the face of prolonged suffering, or to be more reluctant, e.g. in case of assumed ulterior motives of others, or if they were not ready to let go of the person in question themselves. The often reported lower acceptance among physicians compared to the general population might be concealed, as the variable ‘occupation in the health sector’ used in our study was numerically dominated by nurses and other health professionals [40]. The second consistent finding across all models concerned religiosity, confirming results from previous studies [18–20]. Four different frameworks linking religion to attitudes about suicide haven been proposed [26], which might be extended to attitudes towards euthanasia: a moral community context effect, and individual-level religious integration, religious commitment and religious social network. The greatest effect of religiosity showed in the specific scenario of the older cancer patient, the most supported and most consensual case within this study. This suggests that the level of religiosity remains a particularly decisive determinant of rejection in the face of broad, popular support for euthanasia, which in turn is compatible with both commitment to key religious beliefs and integration into religious networks, reinforcing conservative ideologies and separating religious and mainstream culture. Confessional differences in predominantly catholic Austria, on the other hand, hardly seemed to matter—except for Muslims being more restrictive than Catholics in the case of the older cancer patient—but were restricted to differences between those with and those without a formal membership in religious communities, which is compatible with the religious integration perspective [26] and confirms results from other studies [19–20].

A number of empirical correlates emphasised the difference between the three scenarios referring to euthanasia of adults and to the assisted death of the neonate. In contrast to religiosity, the religious confession, for example, did not make a difference in the case of the neonate. Also, whereas a tendency towards a curvilinear effect of SES shows for the abstract scenarios of euthanasia in adults, i.e. both those on the lower and those on the higher end of the social ladder prove less permissive than those in middle positions, the highest 10% of the socio-economic stratification are more approving of the physician-assisted death of the neonate than the lowest 10%. This complex pattern of non-linear relationships regarding socio-economic status, however, is difficult to explain and requires further research.

Differences in attitudes between people living in rural areas, in small towns or in large cities are usually attributed to different value structures, which tend to be more traditional in rural and more liberal in urban settings. Even controlling for a number of attitudes, we still found significant differences. Concerning the abstract and specific scenario of euthanasia for adults, people in cities were more restrictive than their counterparts in villages or smaller towns although the latter did not differ compared to the allegedly more liberal metropolitan area of the capital city. This could hint to a rather ‘pragmatic’ dealing with and an affirmative approach toward euthanasia among the rural population in the face of a potential hospitalisation and continued medical treatment without hope of recovery, neither attributable to liberalism nor to religiosity as both have been controlled for. This is in line with the significant difference found between the rural population and those living in cosmopolitan Vienna regarding physician-assisted suicide and neonatal physician-assisted death, where the latter were more likely in favour of assisted suicide and stronger rejected assisted death of the neonate. In the case of assisted suicide, we reckon that the stigmatised term ‘suicide’ [41] tips the balance in more traditional rural areas. The reversed stronger approval of assisted death of the neonate in less urban areas compared to the capital city might be rooted in the more tolerant and inclusive views on disability and life chances of disabled children in the most urban population. Having heard about the legalisation of euthanasia abroad was associated with a higher general approval of euthanasia but did not extend to the neonate scenario. We reckon this could be either due to self-selection in terms of information processing or refer to positive media coverage of such practices regarding adults in Western European countries, reducing negative associations or stereotypes regarding the issue.

People in larger households, including most likely spouses and cohabiting intergenerational family members, did not show an independent correlation with the rejection of euthanasia but were more likely to reject the assisted death of the neonate. This could be due to a higher familial orientation, commitment for prolonged mutual care and prospective family life in these individuals which relates more the neonate, whose life is still ahead—irrespective of its quality—than to (old age) adults requesting death. The fact that people with small children in the household were more likely to reject assisted suicide but not the other two forms of euthanasia relating to adults, could again be due to the stigmatised notion of the term ‘suicide’ in relation to felt responsibility and to function as role models [42].

Regarding socio-cultural liberalism, the difference between the abstract scenarios on the one hand and the specific scenario of euthanasia (cancer patient) and the specific example of physician-assisted death (neonate) on the other implied that this ideological orientation might only have an effect on abstract notions of these concepts, whereas more immediate emotional responses and social empathy might dominate the approval or rejection in more specific situations of pain and suffering [24]. Empathy is also the keyword in the last predictor tested, authoritarianism. The expected relationship was found for assisted suicide, thereby confirming results from the only previous but small-scale study available [43]. The surprising difference between physician-assisted suicide and the two other forms of euthanasia could be again owing to the term ‘suicide’, which is also visible in the lower overall approval in this scenario compared to euthanasia via medical personnel in the other two cases (see Fig 1). Regarding the severely disabled or ill neonate, our expectation has been partially confirmed. The level of authoritarian personality traits was associated with approval of the neonate’s death but, unlike in the case of assisted suicide, described a non-linear threshold effect for the neonate scenario. The expected effect of a stronger authoritarian personality disposition on an increasing approval of delivering a lethal injection causing the neonate’s death did not hold across the entire spectrum of authoritarianism but varied only within that half of the population, where the personality disposition was in general rather bland (see also Fig 2). Although there seemed to be no difference in terms of approval between high and moderate levels of authoritarianism, the absence of any authoritarian personality disposition was linked to higher rejection rates of physician-assisted death of the neonate. This means more tolerant and rather non-conformist individuals are more likely to reject the assisted death of the severely disabled or ill neonate compared to moderately or strongly conservative and conformist individuals.

## Strengths and Limitations

This study is based on a large, representative national survey providing information on different scenarios of euthanasia and assisted death. Our study assessed associations of attitudes towards euthanasia and assisted death with a comprehensive number of predictors including authoritarianism and explicitly compared determinants across different scenarios. A limitation of this study is the cross-sectional design of the survey, which does not allow inference of causality. Some orientations included were measured by self-rating questions instead of indicators. Regarding religion, future studies should differentiate more clearly between the effects of religious confession, beliefs and networks. Furthermore, not all factors potentially related to attitudes towards euthanasia and assisted death could be included. Particularly values and attitudes regarding autonomy, human dignity, death, the health system and detailed information about episodes of end-of-life-care and death within people’s own social network should be assessed by future research.

## Conclusion

Approval of euthanasia in adults is more likely found in Austria among men than among women of the middle classes, living either in a rather rural context or in the most urban area, who had heard about foreign legalisation, and who can be characterised as non-religious, liberal persons rather than as conformist individuals following traditional authorities. Those approving of the non-voluntary, physician-assisted death of severely ill or disabled neonates were also most likely non-religious, male, of the middle or highest social class, rather live alone, in a non-urban area, are in a poorer state of health and show at least some authoritarian personality disposition. To summarise our findings, we may say on the one hand that there are minor differences in the determinants of rejection among different forms of euthanasia in adults. More substantially, however, we could show that a non-voluntary, physician-assisted death of disabled but non-terminally-ill neonates is approved by a partially different sub-population compared to approval of (voluntary) euthanasia and assisted suicide for adults on the other hand.

## Supporting Information

S1 Dataset Dataset Euthanasia. (SAV)

S1 Table The four scenarios of euthanasia/physician-assisted death used in this study. (DOCX)

S2 Table Authoritarianism: Descriptive statistics and factor loadings. Cronbach’s alpha = 0.79; Kaiser-Meyer-Olkin index = 0.82; principal component analysis with varimax rotation, one factor extracted, explained item variance = 40.95%.
(DOCX)
