# Supplementary material for: Determinants of Public Attitudes towards Euthanasia in Adults and Physician-Assisted Death in Neonates in Austria: A National Survey
Source: PLoS One. 2015 Apr 23;10(4):e0124320. doi: 10.1371/journal.pone.0124320 (PMC4408035; doi:10.1371/journal.pone.0124320)
Supplement: S1 Table — (DOCX) [file pone.0124320.s002.docx]

**Table S1. The four scenarios of euthanasia/physician-assisted death used in the study.**

| Abstract scenario euthanasia:  *‘Do you approve or reject that terminally ill and greatly suffering individuals have their wish to die fulfilled by a medical doctor administering a substance causing their death?’* |
| --- |
| Abstract scenario assisted suicide:  *‘Do you approve or reject that terminally ill and greatly suffering individuals have their wish to die fulfilled by receiving a substance for suicide from qualified personnel?’* |
| Specific scenario euthanasia:  *‘Please assess the following situation: A doctor treats a 79-year old cancer patient who, from a medical perspective, will die from his/her illness. The patient is in great pain and asks the doctor to give him/her an injection, leading to immediate death. Should the doctor fulfil the patient’s wish?’* |
| Specific scenario physician-assisted death:  *‘Please assess the following situation: At the birth of a child, a disease or severe disability is diagnosed, which presumably will leave the child with only few years ahead with poor quality of life. Do you personally approve or reject in this case to administer a lethal substance to the neonate in order to avoid a life full of suffering?* |
