# Supplementary material for: Determinants of Public Attitudes towards Euthanasia in Adults and Physician-Assisted Death in Neonates in Austria: A National Survey
Source: PLoS One. 2015 Apr 23;10(4):e0124320. doi: 10.1371/journal.pone.0124320 (PMC4408035; doi:10.1371/journal.pone.0124320)
Supplement: S2 Table — Cronbach’s alpha = 0.79; Kaiser-Meyer-Olkin index = 0.82; principal component analysis with varimax rotation, one factor extracted, explained item variance = 40.95%. (DOCX) [file pone.0124320.s003.docx]

**Table S2. Authoritarianism: Descriptive statistics and factor loadings.**

| Items | N | Answer categories (%) | | | | Factor loadings |
| --- | --- | --- | --- | --- | --- | --- |
|  |  | agree  completely | rather agree | rather  disagree | disagree completely |  |
| ‘I do not discuss fundamental issues’ | 1854 | 7.7 | 24.7 | 35.1 | 32.5 | .749 |
| ‘If the majority thinks different, I keep my opinion to myself’ | 1906 | 7.2 | 26.2 | 36.3 | 30.3 | .692 |
| ‘People who question established ways upset me’ | 1840 | 6.5 | 25.1 | 36.2 | 32.2 | .683 |
| ‘I feel uncomfortable in new and uncommon situations’ | 1907 | 6.1 | 26.0 | 37.1 | 30.8 | .650 |
| ‘I do not like getting to know new people’ | 1978 | 3.0 | 11.3 | 30.1 | 55.6 | .631 |
| ‘I hardly pity people who are in trouble’ | 1952 | 2.7 | 8.6 | 37.0 | 51.7 | .598 |
| ‘I consider soft people wimps’ | 1929 | 2.5 | 12.8 | 38.5 | 46.2 | .558 |
| ‘I do not mind following orders I do not understand completely’ | 1913 | 8.8 | 25.4 | 35.9 | 29.9 | .529 |

^a^ Cronbach’s alpha = 0.79; Kaiser-Meyer-Olkin index = 0.82; principal component analysis with varimax rotation, one factor extracted, explained item variance = 40.95 %.
